# Investigating *in vitro* and *in vivo* αvβ6 integrin receptor-targeting liposomal alendronate for combinatory γδ T cell immunotherapy

**DOI:** 10.1016/j.jconrel.2017.04.025

**Published:** 2017-06-28

**Authors:** Naomi O. Hodgins, Wafa' T. Al-Jamal, Julie T.-W. Wang, Rebecca Klippstein, Pedro M. Costa, Jane K. Sosabowski, John F. Marshall, John Maher, Khuloud T. Al-Jamal

**Affiliations:** a King's College London, Institute of Pharmaceutical Science, Franklin-Wilkins Building, 150 Stamford Street, London SE1 9NH, UK; b School of Pharmacy, University of East Anglia, Norwich Research Park, Norwich NR4 7TJ, UK; c Barts Cancer Institute, Queen Mary University of London, London EC1M 6BQ, UK; d Centre for Tumour Biology, Barts Cancer Institute, Queen Mary University of London, London EC1M 6BQ, UK; e King's College London, Division of Cancer Studies, Guy's Hospital, London SE1 9RT, UK

**Keywords:** Integrin targeting, Bisphosphonates, γδ T cells, Liposomes, Immunotherapy

## Abstract

The αvβ6 integrin receptor has been shown to be overexpressed on many types of cancer cells, resulting in a more pro-invasive and aggressive phenotype, this makes it an attractive target for selective drug delivery. In tumours that over-express the αvβ6 receptor, cellular uptake of liposomes can be enhanced using ligand-targeted liposomes. It has previously been shown in both *in vitro* and *in vivo* studies that liposomal alendronate (L-ALD) can sensitise cancer cells to destruction by Vγ9Vδ2 T cells. It is hypothesised that by using the αvβ6-specific peptide A20FMDV2 as a targeting moiety for L-ALD, the therapeutic efficacy of this therapy can be increased in αvβ6 positive tumours. Targeted liposomes (t-L) were formulated and the targeting efficacy of targeted liposomes (t-L) was assessed by cell uptake and cytotoxicity studies in the αvβ6 positive cells line A375Pβ6. Bio-distribution of both L and t-L were carried out in αvβ6 positive (A375Pβ6 and PANC0403) and αvβ6 negative (A375Ppuro and PANC-1) subcutaneous tumour mouse models. Immuno-compromised mice bearing A375Pβ6 experimental metastatic lung tumours were treated with L-ALD or t-L-ALD as monotherapies or in combination with *ex vivo*-expanded Vγ9Vδ2 T cells. *In vitro*, αvβ6-dependant uptake of t-L was observed, with t-L-ALD being more effective than L-ALD at sensitising A375Pβ6 to γδ T cells. Interestingly, t-L-ALD led to slightly higher but not significant reduction in tumour growth compared to L-ALD, when used as monotherapy *in vivo*. Moreover, both L-ALD and t-L-ALD led to significant reductions in tumour growth when used in combination with γδ T cells *in vivo* but t-L-ALD offered no added advantage compared to L-ALD.

## Graphical abstract

Formulation of αvβ6 targeted liposomes. Liposomes were formulated with 1% DSPE-PEG2000 Maleimide (%mol/total lipid). The peptide A20FMDV2 was modified to contain a cysteine residue at the C-terminus. These liposomes were incubated with 25 μg peptide/μmol lipid of the peptide A20FMDV2 overnight at RT. The thiol group of the C-terminus cysteine residue reacts with the maleimide group to form a thioether bond.

**Figure:**
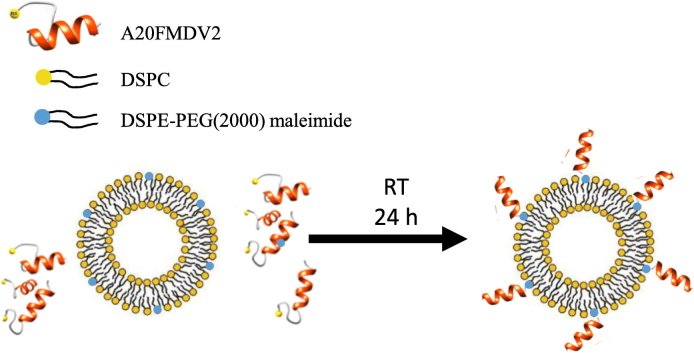

## 1 Introduction

Integrins are heterodimeric glycoproteins composed of non-covalently linked α and β subunits and are involved in a variety of cell processes including proliferation [1], survival [2], migration [3] and invasion [4], giving them a key role in cancer. The αvβ6 integrin receptor is expressed on epithelia, usually only in the process of tissue remodelling [5] and wound healing [6]. However, overexpression of this receptor has been detected in many types of tumours including colon cancer [7], gastric carcinomas [8], oral squamous cell carcinomas [9] and breast cancer and is typically associated with a more pro-invasive and aggressive phenotype [10], [11]. Therefore, this integrin provides an attractive molecular target for targeted drug delivery to cancer cells. The 20 amino acid peptide, A20FMDV2, (NAVPNLRGDLQVLAQKVART), derived from the VP1 coat-protein of the foot-and-mouth disease virus, has shown very good specificity for αvβ6 [12]. This peptide has been shown to effectively and specifically target αvβ6-expressing cancers [13]. A20FMDV2 has not previously been conjugated to any type of nanocarrier and its ability to target nanoformulations has not been tested. Only one other study has targeted the αvβ6 integrin receptor using a nanoparticle formulation. In that study, liposomes were targeted using an alternative peptide, H2009.1 [14].

Alendronate (ALD), is a nitrogen containing bisphosphonate (N-BP) that can sensitise tumour cells to killing by Vγ9Vδ2 T cells in both *in vitro* [15], [16], [17], [18], [19] and *in vivo* studies [20], [21], [22], [23], [24], [25], [26], [27]. The encapsulation of ALD in liposomes (L-ALD), has been shown to increase its therapeutic efficacy [24]. Long-circulating liposomes passively target the tumour due to the enhanced permeation and retention (EPR) effect [28], leading to a greater amount of the encapsulated drug reaching the tumour cells.

The aim of this study is to formulate αvβ6 integrin targeted ALD liposomes (t-L-ALD), using the peptide A20FMDV2. It is hypothesised that A20FMDV2 conjugation to liposomal alendronate will promote αvβ6-receptor mediated endocytosis and improved therapeutic efficacy in combination with γδ T cell immunotherapy *in vitro* and possibly *in vivo*.

## 2 Materials and methods

Additional methods are described in Supplementary information.

### 2.1 Materials

1,2-distearoyl-*sn*-glycero-3-phosphocholine (DSPC) and 1,2-dipalmitoyl 1,2-distearoyl-*sn*-glycero-3-phosphoethanolamine-N-[methoxy(polyethylene glycol)-2000] (ammonium salt) (DSPE-PEG2000) were obtained from Lipoid (Germany). 1,2-dioleoyl-*sn*-glycero-3-phosphoethanolamine-N-(carboxyfluorescein) (CF-DOPE), 1,2-distearoyl-*sn*-glycero-3-phosphoethanolamine-N-diethylenetriaminepentaacetic acid (ammonium salt) (DSPE-DTPA) and DSPE-PEG(2000) maleimide were purchased from Avanti Polar Lipids, Inc. (USA). The cysteine-modified A20FMDV2 peptide (Sequence: NAVPNLRGDLQVLAQKVART-Cysteine, Purity: > 95%) was purchased from Genscript Limited (Hong Kong). Dextrose, cholesterol, sodium chloride, phosphate buffered saline (PBS) tablets, N-(2-Hydroxyethyl)piperazine-N′-(2-ethanesulfonic acid) (HEPES), methanol (Analytical reagent grade), chloroform (Analytical reagent grade), and Sepharose 2B were purchased from Sigma (UK). LavaPep™ Protein and Peptide quantification kit was obtained from Web Scientific (UK). PD-10 desalting column was obtained from GE Healthcare Life Sciences (UK). Snake Skin® dialysis tubing (MWCO 10000 Da) was purchased from Thermo-fisher (USA). Dulbecco's modified Eagle's medium (DMEM), Roswell Park Memorial Institute medium (RPMI), Glutamax™ and antibiotic-antimycotic solution were purchased from Invitrogen (UK). Foetal Bovine Serum was purchased from First Link (UK). Human AB serum (male) was obtained from Sigma (UK). Thiazolyl blue tetrazolium bromide (MTT), tropolone and alendronate sodium trihydrate were obtained from Alfa Aesar (UK). DMSO was obtained from Fisher (UK). Human IFN-γ ELISA Ready-set-go kit was purchased from eBiosciences (UK). The 10D5 antibody was obtained from Abcam (UK). FITC labelled secondary antibody was purchased from Cell Signalling (UK). D-Luciferin was obtained from Perkin Elmer (UK). Indium-111 chloride was obtained from Mallinckrodt (NL). Thin layer chromatography (TLC) strips for radio-labelling were purchased from Agilent Technologies UK Ltd. (UK). Isoflurane (IsoFlo®) for anaesthesia was purchased from Abbott Laboratories Ltd. (UK). All reagents were used without further purification.

### 2.2 Preparation of liposomes

Stock solutions of 40 mg/ml were prepared in chloroform/methanol (4:1 v/v). To avoid degradation, lipid solutions were stored at − 20 °C under nitrogen. Untargeted Liposomes (L) were prepared by thin film hydration (TFH). DSPC, cholesterol and DSPE-PEG2000 (55:40:5 molar ratio) were added to a 25 ml round-bottom flask and 2 ml chloroform/methanol (4:1 v/v) was added. A thin lipid film was formed upon removal of the solvent under reduced pressure using a rotary evaporator (Rotavapor® R-210, Buchi UK). The lipid film was flushed with nitrogen to remove any remaining traces of organic solvent. The film was then hydrated by adding 1 ml of PBS, adjusted to pH 7.4. The liposome suspension was left for 1 h at 60 °C and was vortexed (Vortex genie 2, Scientific Industries Inc., USA) every 15 min [29]. Liposomes were prepared at a final concentration of 25 mM total lipid. The size and polydispersity (PDI) of the liposomes was reduced with serial extrusion using the mini-extruder (Avanti Polar Lipids, USA) through polycarbonate membranes (Avanti Polar Lipids, USA) with pore sizes 0.8 μm (5 ×), 0.2 μm (5 ×), 0.1 μm (10 ×) and 0.08 μm (15 ×) at 60 °C. The resulting suspension was stored at 4 °C.

For targeted liposomes (t-L), the liposomes were formed as above with the addition of 0.5–2%mol of DSPE-PEG_2000_-maleimide. The overall formulation of the liposomes was DSPC:cholesterol:DSPE-PEG_2000_:DSPE-PEG_2000_-maleimide (55:40:3–4.5:0.5–2 molar ratio). The DSPE-PEG_2000_ was reduced in order to keep the overall percentage of PEG2000 at 5%mol. After the liposomes were formed, they were flushed with nitrogen and incubated with A20FMDV2 peptide at RT overnight (25–100 μg peptide/μmol lipid) under nitrogen. Excess peptide and ALD was removed by eluting the liposomes through a Sephadex 2B column with PBS; or *via* overnight dialysis against PBS using a dialysis bag with a MWCO of 10,000 kD at room temperature.

For cellular uptake studies, fluorescent liposomes were formed as above but with the inclusion of 1% mol CF-DOPE to give a final liposome composition of DSPC:CF-DOPE:cholesterol:DSPE-PEG_2000_:DSPE-PEG_2000_-maleimide (54:1:40:4:1 molar ratio).

Liposomes containing alendronate (L-ALD and t-L-ALD) were prepared as above, but the lipid film was hydrated with 1 ml of 100 mM solution of ALD in HEPES Buffered Saline (HBS, 20 mM HEPES, 150 mM NaCl). Un-encapsulated ALD was removed by overnight dialysis against HBS using a dialysis bag with a MWCO of 10,000 kD.

### 2.3 Peptide quantification

The amount of peptide conjugated to the liposomes was determined by LavaPep™ Protein and Peptide quantification kit. A calibration curve was obtained in the range 0.122–500 μg/ml using free A20FMDV2. Liposomes were diluted 100 times in deionised water and the amount of peptide quantified according to the manufacturer's instructions. Briefly, 50 μl of the diluted sample was incubated with 50 μl of LavaPep working solution for 60 min in the dark at RT. The fluorescence intensity was then measured using 540 ± 10 nm and 630 ± 10 nm excitation and emission filters, respectively (FLUOStar Omega, BMG Lab Tech). The per cent peptide conjugated to the liposomes was calculated by quantifying the amount of peptide in the liposome sample before and after purification.

### 2.4 Cell culture conditions

The cell lines PANC-1 (CRL-1469™, pancreatic), PANC0403 (CRL-2555™, pancreatic) and 4T1 (CRL-2539™, breast) were obtained from ATCC®. A375Ppuro and A375Pβ6puro cell lines were created using the human melanoma cell line A375P (CRL-3224™, melanoma), which was infected with pBabe retroviruses encoding puromycin resistance alone or in combination with cDNA for human β6, as previously reported [12]. The A375Ppuro and A375Pβ6 cell lines were a kind gift from Prof. John Marshall (QMUL). The A375Pβ6 cell line was subsequently transfected with firefly luciferase (luc) using an SFG retroviral vector whereby luc was co-expressed with dsTomato red fluorescent protein. Transduced cells were then flow sorted for red fluorescence to obtain a pure A375Pβ6-luc cell line [24]. All cell lines were maintained at 37 °C, 5% CO_2_ and 5% relative humidity. Advanced RPMI (PANC-1, PANC0403, 4T1) or DMEM media (A375Ppuro, A375Pβ6puro) were used, both of these were supplemented with 10% FBS, 1% GlutaMAX™ and 1% Penicillin/Streptomycin.

### 2.5 Characterisation of cell lines for αvβ6 integrin expression

αvβ6 integrin receptor expression was confirmed by 10D5 antibody staining and flow cytometry. Cells (1 × 10^5^/100 μl) were incubated with 5 μl of 10D5 or the isotype control (IgG FITC) for 30 min at 4 °C, washed twice with 1 ml PBS before 30 min incubation with 2.5 μl of the FITC labelled IgG secondary antibody at 4 °C then washed with PBS. Using the FL1 detector, 10,000 cells were gated and the fluorescence was analysed under live gating. The cells were read on a BD FACS Calibur™ flow cytometer obtained from BD Bioscience (US) and analysed using FlowJo software.

### 2.6 Cellular uptake of liposomes using flow cytometry

Cells were plated in a 24 well plate at a density of 50,000 cells/well and left overnight to allow the cells to attach. Cells were treated with 32.5–130 μM CF-DOPE containing liposomes (L or t-L) in complete media (10% FBS) for 1 or 4 h. In order to determine if the increased uptake of t-L was αvβ6 receptor specific, peptide inhibition studies were carried out. Cells were incubated at 4 °C for 10 min and were then treated with 0.2 ml of 50 μg/ml free peptide in complete media for a further 10 min on ice. The fluorescently labelled L and t-L (32.5–130 μM) were then additionally incubated with the cells for 1 or 4 h. Additional peptide (50 μg in 25 μl PBS) was added after 1 h to ensure that the αvβ6 receptors remained blocked. Upon completion of the incubation period, the cells were washed with PBS, trypsinised and transferred into BD flow cytometer tubes. The cells were washed in PBS and re-suspended in 500 μL of PBS. The fluorescence was analysed in triplicates for each condition using the FL1 detector with 10,000 cells gated. All flow cytometry data was acquired using a BD FACS Calibur™ flow cytometer obtained from BD Bioscience (US) and analysed using FlowJo software.

### 2.7 Treatment of cancer cell lines with L-ALD/t-L-ALD and γδ T cells in combination therapy studies

The cell lines A375Ppuro, A375Pβ6, PANC-1 and PANC0403 were seeded at 50,000 cells/well in 96 well plates. Cells were treated for 24 h with 30 or 60 μM of ALD, L-ALD or t-L-ALD. Empty-liposomes (EL) and targeted EL (t-EL) were used as controls at lipid concentrations equivalent to that of the L-ALD and t-L-ALD, depending on the drug loading. The treatments were removed and replaced with 2.5 × 10^5^ *ex vivo* expanded Vγ9Vδ2 T cells, or γδ T cell culture media as a control, for a further 24 h. The γδ T cells were then removed and the cell monolayers were washed with PBS before cell viability was assessed with MTT as described below.

### 2.8 MTT assay

MTT (3-(4,5-dimethylthiazol-2-yl)-2,5-diphenyltetrazolium bromide) solution was prepared in PBS at a concentration of 5 mg/ml and was diluted in media (1:6) prior to use. The supernatant of each well was removed and MTT solution (120 μl) was added to each well. The plates were then incubated at 37 °C and 5% relative humidity for 3 h. The MTT solution from each well was removed and DMSO (200 μl/well for 96 well) was added and incubated for 5 min at 37 °C, to eliminate air bubbles. The absorbance was read at 570 nm with reference at 630 nm (FLUOStar Omega, BMG Lab Tech). Percentage cell survival was determined by calculating the absorbance of treated cells as a percentage of that of untreated cells.

### 2.9 Determination of IFN-γ concentration by ELISA

Supernatant from the co-culture assay was removed from each of the wells immediately before the cytotoxicity assay was performed. The supernatant was centrifuged to remove the γδ T cells and was stored at − 80 °C until required. Supernatants were diluted 1:40 and analysed using a human IFN-γ ELISA Ready-set-go-kit as per the manufacturer's protocol.

### 2.10 Radiolabelling of DTPA containing liposomes

DTPA containing liposomes were prepared with the TFH method as above but with 1% of the DSPC replaced with 1% molar ratio of DSPE-DTPA, and radiolabelled with ^111^In [30]. The required volume of ^111^In, containing 1 MBq per mouse for bio-distribution studies or 10–15 MBq per mouse for imaging studies was added to 2 M ammonium acetate buffer (one-ninth of the reaction volume, pH 5.5). This was then added to the liposome sample (100 μl of 20 mM liposomes/mouse) to give a final ammonium acetate concentration of 0.2 M, and incubated for 30 min at RT. The reaction was quenched by the addition of 0.1 M EDTA solution to the mixture (5% v/v of the reaction mixture) to chelate free ^111^In. Unbound ^111^In:EDTA was removed using NAP-5 desalting columns equilibrated with PBS and the liposomes were collected in fraction 1–3 (~ 150 μl per injection dose).

### 2.11 Efficiency and stability of the radiolabelling in serum

Samples of the radiolabelled liposomes or ^111^In:EDTA were spotted in glass microfibre chromatography paper impregnated with silica gel. These strips were then developed using a mobile phase of 50 mM EDTA in 0.1 M ammonium acetate. Strips were placed on a multi-purpose storage phosphor screen (Cyclone®, Packard, Japan) and kept in an autoradiography cassette (Kodak Biomax Cassette®) for 10 min. Quantitative autoradiography counting was then carried out using a cyclone phosphor detector (Packard®, Australia). The labelling stability was tested by incubation of the radio-conjugates in the presence or absence of FBS. Samples were diluted in 50% FBS or PBS [1:2 (v/v)], and incubated for 24 h at 37 °C. The percentage of ^111^In:EDTA (immobile spot) remained conjugated to the liposomes was evaluated by TLC, using the same protocol as described above.

### 2.12 Animal models

All animal experiments were performed in compliance with the UK Home Office (1989) Code of Practice for the housing and care of Animals used in Scientific Procedures. Female SCID/Beige (SPECT/CT studies) and male NOD SCID gamma (NSG) mice (bio-distribution and therapy studies), 4–6 weeks old, were obtained from Charles River (UK). Female BALB/c mice, 4–6 weeks old, were obtained from Harlan Laboratories (UK). For the human xenograft tumour models, subcutaneous (s.c.) tumours were established by injecting 5 × 10^6^ cells in 100 μl PBS into each of the rear-flanks of SCID/Beige. For the murine tumour model, 1 × 10^6^ cells in 100 μl PBS were injected into each of the rear flanks of BALB/c mice. The size of the tumour was measured using callipers and tumour volume were determined using the equation:

$$\text{Tumour Volume}\left({\mathrm{mm}}^{3}\right)=\left(A^{2}\mathrm{B\pi}\right)/6$$

where *A* and *B* represent the width and the length of the tumours, respectively [31]. Experiments commenced when tumours reached ~ 300 mm^3^. For the lung model, 5 × 10^5^ cells were injected into the tail vein of NSG mice. This tumour model was monitored *via* s.c. injection of the mice with 100 μl 30 mg/ml luciferin/20 g mouse and subsequent scanning after 20 min using an IVIS Lumina series III *In Vivo* Imaging system (Perkin-Elmer). Images were quantitatively analysed by drawing regions of interest (ROI) around the tissues using Living Image 4.3.1 Service Pack 2 software (Perkin-Elmer, USA).

### 2.13 Whole body SPECT/CT imaging of radiolabelled liposomes in tumour-bearing mice

Mice were injected with liposomes containing 2 μmol lipid and radiolabelled with 1 MBq or 10–15 MBq, for bio-distribution and SPECT/CT studies, respectively, *via* tail vein injection. Mice were imaged with nanoSPECT/CT scanner (Bioscan ®, USA) 0–30 min, 4 h and 24 h post i.v. injection. For each mouse, a tomography was initially performed (45 Kvp; 1000 ms) to obtain parameters required for the SPECT and CT scanner, including the starting line, finish line and axis of rotation of the acquisition for each mouse. SPECT scans were obtained using a 4-head scanner with 1.4 mm pinhole collimators and the following settings: number of projections: 24; time per projection: 60 s and duration of the scan 60 min. CT scans were obtained at the end of each SPECT acquisition using 45 Kvp. All data were reconstructed with MEDISO (medical Imaging System) and the combining of the SPECT and CT acquisitions were performed using PMOD® software.

### 2.14 Gamma counting of radiolabelled liposomes in tumour-bearing mice

After 24 h, the mice were perfused with ~ 25 ml of 1000 U/l heparin in 0.9% sodium chloride in order to remove any liposomes remaining in the blood. The major organs (brain, lung, liver, spleen, kidney, heart, stomach and intestine), muscle, skin, bone (femur), carcass and tumours were collected, weighed and placed in scintillation vials. Additionally, 5 μl blood samples taken at various time points (5, 10, 30, 60, 240 and 1440 min), and urine and faeces collected with the aid of metabolic cages were also placed in scintillation vials for analysis. Each sample was analysed for [^111^In] specific activity using an automated gamma counter (LKB Wallac 1282 Compugamma, PerkinElmer, UK) together with dilutions of injected dose with dead time limit below 60%. The gamma rays emitted by the radioisotope were detected, quantified and corrected for physical radioisotope decay by the gamma counter. Radioactivity readings (counts per minute - CPM) were plotted as percentage of injected dose per organ or percentage of injected dose per gram of tissue.

### 2.15 Therapy study

Male NSG mice (4–6 weeks) were inoculated with 5 × 10^6^ A375Pβ6.luc cells by i.v. injection to form experimental metastatic lung tumours. Bioluminescence imaging of mice was carried out on day 6 as described above and mice were divided into 4 treatment groups: naïve, L-ALD, γδ T cells and L-ALD and γδ T cells combination treatment. Doses used in therapy experiments were 0.5 μmol of ALD/mouse (L-ALD) and 1 × 10^7^ cells/mouse (γδ T cells), all injected *via* the tail vein. Three doses of each treatment were given at one-week intervals on dates 7, 14 and 21. In the case of the combination treatment, mice were pre-injected with L-ALD (days 6, 13, and 20) then injected with γδ T cells (days 7, 14, and 21). Tumour growth was monitored by bioluminescence imaging twice weekly, as described above.

### 2.16 Determination of IFN-γ concentration with ELISA

Animals from the therapy study were sacrificed and sera was analysed for human IFN-γ. Sera were diluted 1:2 and analysed using a human IFN-γ ELISA Ready-set-go-kit as per the manufacturer's protocol.

### 2.17 Pre-incubation of liposomes with mouse sera

Mouse serum was obtained by allowing blood obtained from a terminal bleed of SCID/Beige mice to clot. The blood was centrifuged to pellet the clotted cells, and the serum was removed for further use. t-L were incubated with the mouse serum for various periods of time (10 min, 1 h, 4 h) at 37 °C. Any proteins that had not interacted with the surface of the liposomes were removed using a 10 cm Sepharose 2B column and eluted using PBS. t-L were incubated with cells without any further processing and their uptake analysed by flow cytometry as described above.

### 2.18 Statistics

For all experiments, data were presented as mean ± SD, except for therapy experiments where data were presented as mean ± SEM; *n* denotes the number of repeats. Independent variable Student *t*-tests were performed using IBM SPSS version 20 for *in vitro* cytotoxicity studies. For *in vivo* studies, significant differences were examined using one-way ANOVA. The t-value, degrees of freedom and two-tailed significance (*p*-value) were determined. *p < 0.05, **p < 0.01 and ***p < 0.001.

## 3 Results

### 3.1 Preparation and characterisation of αvβ6 integrin-targeted liposomes (t-L)

DSPC:Chol:DSPE-PEG_2000_ liposomes were prepared using lipid film hydration method and extrusion. t-L were prepared by incorporating DSPE-PEG_2000_-maleimide (0.5–2 mol%) in the liposomes, then linked directly to αvβ6-targeting peptide. Free peptide was removed before further characterisation. The effects of %mol DSPE-PEG_2000_-maleimide and the peptide A20FMDV2 (25 μg/μmol lipid) on the physicochemical characteristics of the liposomes were examined. There was no significant difference in the hydrodynamic size (157.8–159.1 nm), PDI (0.080–0.098) or zeta potential (− 12.5 to − 14.6 mV) of the liposomes when DSPE-PEG_2000_-maleimide (1 mol%) or A20FMDV2 were included in the formulation (Table 1). The size of the liposomes is in the range reported to be extravasated in regions of leaky vasculature as part of the EPR effect [32]. The low PDI values indicated that the liposomes were homogenous in size and the absence of liposome aggregation. The identical physicochemical characteristics of the L and t-L ensure that any differences in behaviour of these liposomes are solely due to the ability of the A20FMDV2 peptide to bind to the αvβ6 integrin receptor. Liposomes containing 0.5 or 2% DSPE-PEG_2000_-maleimide with or without A20FMDV2 were also characterised and no significant change to the physicochemical characteristics of the liposomes were observed (Table S1).

**Table 1:** Physicochemical characteristics of liposomes with or without peptide.

Initial peptide added (μg/μmol lipid)^a^	Size (nm)^b^, ^d^	PDI^b^, ^d^	Zeta Potential (mV)^c^, ^d^
0	159.1 ± 1.7	0.098 ± 0.025	− 14.6 ± 1.53
25	157.8 ± 2.5	0.08 ± 0.013	− 12.5 ± 1.08

a Liposomes containing 1% mol DSPE-PEG_2000_-maleimide.

b Hydrodynamic diameter measured by dynamic light scattering.

c Analysed by electrophoretic light scattering using 10 mM NaCl.

d Data are represented as mean ± SD.

### 3.2 Optimisation of peptide loading on liposomes surface

The effect of different initial addition of peptide on the final peptide loading on the t-L was examined. The LavaPep peptide assay was used to quantify the amount of peptide conjugated to the t-L and confirmed the purification of the t-L from the unconjugated A20FMDV2 using a Sepharose 2B column (Fig. S1). In the case of 1%mol DSPE-PEG_2000_-maleimide, the amount of A20FMDV2 added to the liposomes was adjusted from 25 to 100 μg per μmol lipid (10–40 nmol per mol lipid) (Table 2). Final peptide loading achieved ranged from 17.7 to 21.7 μg peptide/μmol lipid. There was no significant difference between the amount of A20FMDV2 added and the final peptide loading so it was therefore decided to use 25 μg peptide per μmol lipid (initial peptide loading) to prepare the t-L for subsequent studies. Additionally, when the %mol DSPE-PEG_2000_-maleimide was adjusted (0.5 or 2 mol%), the amount of peptide loaded onto the liposomes increased proportionally to the % of DSPE-PEG_2000_-maleimide included in the liposome formulation (Table S2). All t-L used for *in vitro* and *in vivo* experiments contained 1% mol DSPE-PEG_2000_-maleimide, as initial experiments determined that no improvement in targeting efficacy was observed when the DSPE-PEG_2000_-maleimide content was increase to 2% mol (Figs. S2 and S3).

**Table 2:** Optimisation of peptide loading on liposomes.

Initial peptide added (μg/μmol lipid)^a^	Final peptide loading (μg/μmol lipid)^b^, ^d^	nmol peptide/μmol lipid^b^, ^d^	% Peptide conjugated^c^, ^d^
25	21.7 ± 4.5	8.5 ± 1.8	84.8 ± 17.6
50	17.7 ± 4.6	6.9 ± 1.8	34.5 ± 8.9
100	21.2 ± 0.9	8.3 ± 0.4	20.67 ± 0.9

a Liposomes containing 1% mol DSPE-PEG_2000_-maleimide.

b Determined by LavaPep Peptide quantification kit.

c Calculated as a percentage of initial peptide added.

d Data are represented as mean ± SD.

### 3.3 *In vitro* targeting of t-L is αvβ6 integrin expression dependant

The targeting efficacy of the liposomes was examined by looking at the uptake of fluorescently-labelled L and t-L in αvβ6 positive (A375Pβ6, 4T1 and PANC0403) and negative (A375Ppuro and PANC-1) cell lines. The results are displayed as mean fluorescence intensity (MFI) ratio of t-L to L. When a value of 1 is obtained the uptake of L and t-L were the same. A value of > 1 indicates that the t-L was taken up in greater quantities than L, and that a positive targeting effect has occurred. All experiments were carried out in media containing 10% foetal bovine serum (FBS), as the large increase in internalisation of both L and t-L by starved cells in serum-free media masked any targeting effect of t-L (Fig. S4).

**Fig. 1:**
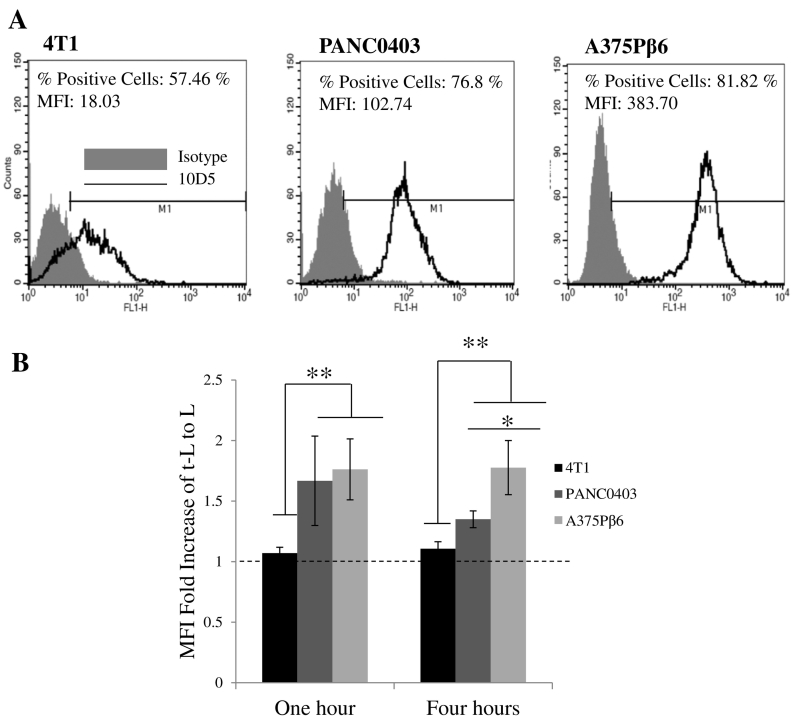
The effect of the extent of expression of αvβ6 integrin receptor on the uptake of t-L. (A) Representative images of cell lines incubated with the antibody 10D5 (specific to αvβ6) or an isotype control. Cells were analysed for αvβ6 integrin receptor expression using flow cytometry and FL-1 detector. In a comparison of the αvβ6 expression of different cell lines using the % positive cells gated in M1 or the MFI, the 4T1 cancer cell line showed the lowest receptor expression, while A375Pβ6 has the highest expression both in terms of % of positive cells and the number of receptors per cell. (B) Comparison of the MFI fold increase of fluorescently labelled t-L to L. Cells were treated with 32.5 μM L or t-L for 1 or 4 h. With increasing expression of αvβ6, the uptake of t-L in relation to L further increases. This is a further indication of the specificity of the t-L to the αvβ6 integrin receptor. Values are expressed as mean ± SD (*n* = 3). *p < 0.05, **p < 0.01 (ANOVA).

To evaluate the targeting efficiency of t-L, three αvβ6 integrin positive cell lines were used. These were the αvβ6 integrin-transfected human melanoma cell line A375Pβ6 and two cell lines that naturally express αvβ6: the human pancreatic cancer cell line, PANC0403 and the murine breast cancer cell line 4T1. The antibody 10D5 was used to measure the expression of αvβ6 integrin receptor on the panel of cancer cell lines used. As seen in Fig. 1, 4T1 cells expressed the lowest amount of αvβ6 integrin receptor, while higher expression was seen in PANC0403 and A375Pβ6 cells. A correlation between the extent of αvβ6 integrin receptor expression on the surface of cells and the fold increase of t-L uptake compared to L could be seen. A375Pβ6 and PANC0403 showed a significantly higher fold increase of t-L/L compared to 4T1 (*p* < 0.01) at both 1 and 4 h. Additionally, at 4 h, A375Pβ6 demonstrated enhanced uptake of t-L/L compared to PANC0403 (*p* < 0.05). These data demonstrate the αvβ6 integrin-specific targeting effect of the liposomes. The results also suggest that a certain level of expression of the receptor is required for targeting to occur.

### 3.4 Uptake of t-L in αvβ6 expressing cells is inhibited with the free peptide A20FMDV2

**Fig. 2:**
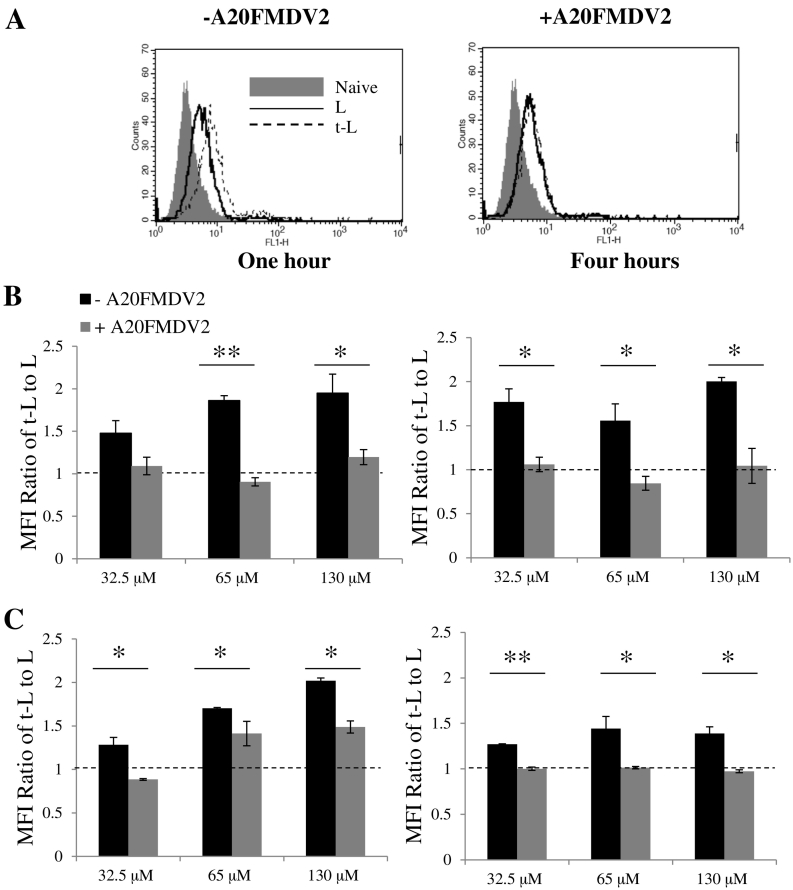
Cellular uptake of fluorescent liposomes in αvβ6 positive cancer cell lines *in vitro*. A375Pβ6 and PANC0403 cells were incubated with L or t-L labelled with 1 mol% *CF*-DOPE for either 1 or 4 h. Cells were pre-treated on ice with either normal media (black bars) or media containing 50 μg/ml (grey bars) of the free A20FMDV2 peptide for 10 min prior to addition of the liposomes. Cellular uptake was assessed by measuring the mean fluorescence intensity (MFI) using flow cytometry (A) Flow cytometry plots demonstrate the reduction in t-L uptake in A375Pβ6 cells in the presence of free A20FMDV2. (B) A375Pβ6 showed reduced uptake of t-L when incubated with free A20FMDV2 at both 1 and 4 h. (C) Similarly, PANC0403 cells also showed a reduction in targeting ability of t-L when incubated with free A20FMDV2. *p < 0.05, **p < 0.01 (Student's *t-*test + A20FMDV2 *vs.* –A20FMDV2).

To further prove that enhanced liposomal targeting efficiency was related to the interaction between the peptide in t-L and the αvβ6 integrin receptor, peptide inhibition studies were carried out in the presence of excess A20FMDV2. Fig. 2 displays the results obtained from the melanoma and pancreatic cancer paired cell lines. A 1.5- to 2-fold increase in the uptake of t-L compared to L was seen at both 1 and 4 h for the αvβ6 positive cell line, A375Pβ6 (Fig. 2B). However, enhancement of liposomal uptake was significantly reduced in the presence of the free A20FMDV2 (*p* < 0.05) with a ratio of ~ 1 obtained when compared with untargeted liposomes. A similar trend was observed for the αvβ6 positive PANC0403 cell line, whereby, when the cells were incubated with A20FMDV2, a lowering of the fold increase in liposomal uptake could be seen (*p* < 0.05) (Fig. 2C). This further indicates that the increased amount of t-L taken up by the cells in relation to L is αvβ6 receptor-dependent. The αvβ6 negative cell lines, A375Ppuro and PANC-1, (Fig. S5) were also used to further confirm the specificity of the t-L. PANC-1 took up equal amounts of L and t-L and this did not change in the presence of free A20FMDV2. For A375Ppuro, a small increase in the amount of t-L when compared to L was observed in some conditions and was affected by the presence of A20FMDV2, despite their lack of the integrin receptor. This t-L/L ratio was lower than that of αvβ6 positive cell lines, however, and only occurred sporadically.

### 3.5 t-L-ALD are more effective than L-ALD at sensitising an integrin positive cell line to destruction by γδ T cells

**Fig. 3:**
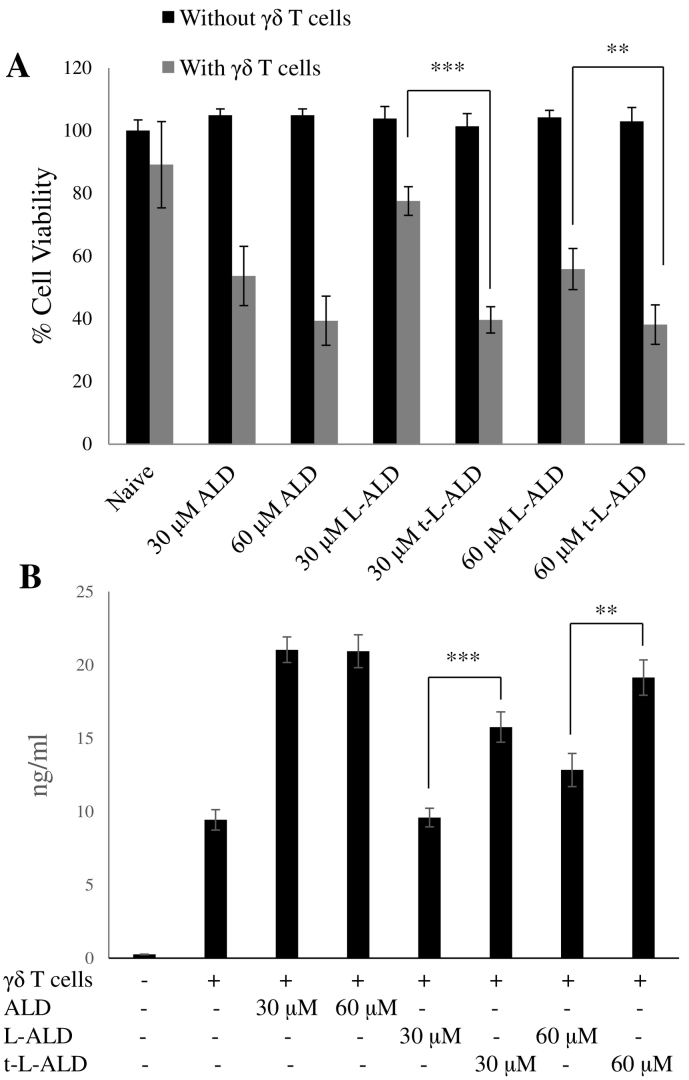
The ability of L-ALD and t-L-ALD to activate Vγ9Vδ2 T cells. (A) Cells were treated with ALD, L-ALD or t-L-ALD for 24 h at 30 or 60 μM for 24 h. The treatments were then removed and replaced with 2 × 10^5^ γδ T cells for a further 24 h before a MTT assay was performed. t-L-ALD increased the sensitivity of A375Pβ6 cells to γδ T cells compared to L-ALD (grey bars). ALD, L-ALD or t-L-ALD did not cause cytotoxicity alone at the concentrations used (black bars). (B) IFN-γ ELISA was performed on supernatant removed prior to the MTT assay. t-L-ALD led to higher release of IFN-γ from γδ T cells than L-ALD. Data was expressed as means ± SD (*n* = 5). **P < 0.01, ***P < 0.001, (Student's *t-*test L-ALD *vs.* t-L-ALD).

In order to test the therapeutic benefit of using liposomes targeted with the A20FMDV2 peptide, ALD was encapsulated into L and t-L formulations. The ability of the resulting L-ALD and t-L-ALD to sensitise cancer cell lines to destruction by Vγ9Vδ2 T cells was then tested by assessing cell viability and γδ T cell-derived IFN-γ production. The αvβ6 positive cell line A375Pβ6 was used in this assay. As shown in Fig. 3, none of the treatments in isolation caused toxicity. However, when the cells were pre-treated with free or liposomal ALD, and were subsequently treated with γδ T cells, a significant decrease in cell viability was observed. t-L-ALD in combination with γδ T cells led to significantly lower cell viability than L-ALD at both 30 μM (*p* < 0.001) and 60 μM (*p* < 0.01) concentrations. To further confirm the increased sensitivity of αvβ6 positive cancer cells to γδ T cells when treated with t-L-ALD as compared to L-ALD, the IFN-γ release from the γδ T cells was quantified. Significantly higher amounts of IFN-γ were released when the γδ T cells were co-cultured with cells pre-treated with t-L-ALD as compared to L-ALD (*p* < 0.001 and *p* < 0.001 for 30 μM and 60 μM, respectively). This finding is in agreement with the results obtained by the MTT assay. In the case of the αvβ6 negative cell line, A375Ppuro, no difference in the ability of L-ALD and t-L-ALD to sensitise the cells to γδ T cells was seen in terms of either cytotoxicity or IFN-γ release (Fig. S6).

### 3.6 Whole body SPECT/CT imaging of mice injected with ^111^In labelled L and t-L

L and t-L were formulated with 1% mol DSPE-DTPA and radiolabelled with ^111^In. Labelling efficiencies of 86.3% and 61.9% were obtained for L and t-L, respectively (Fig. S7). Stability studies were performed after 24 h at 37 °C in both PBS and 50% FBS in order to replicate *in vivo* conditions. For L, 87.8% of the ^111^In remained bound to the liposomes after 24 h incubation with PBS. On incubation of L with serum, 91% stability was seen. For t-L, 80.7% and 78.2% of ^111^In remained bound after 24 h incubation with PBS and FBS, respectively.

**Fig. 4:**
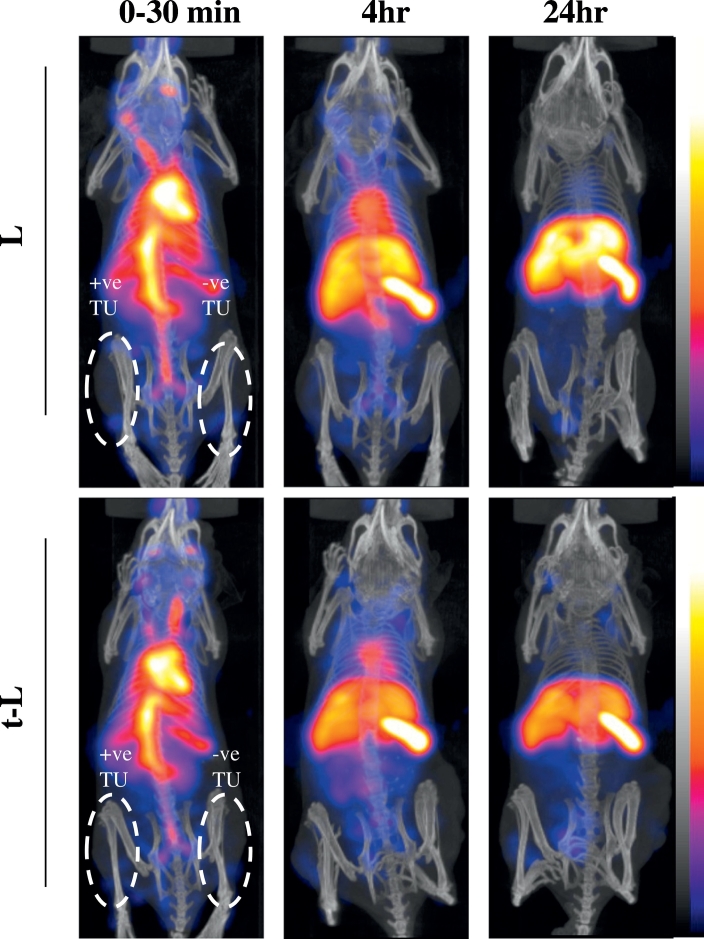
*In vivo* whole body 3D SPECT/CT imaging of ^11^In labelled liposomes in tumour-bearing immunocompromised mice. Mice were i.v. injected with ^111^In labelled liposomes at a dose of 2 μmol lipid/mouse. Mice were inoculated bi-focally with the αvβ6 positive tumour on the left of the image (A375Pβ6) (right flank) and the negative tumour (A375Ppuro) on the right of the image (left flank). Whole body 3D SPECT/CT imaging were performed at 0–30 min, 4 and 24 h post-injection with scanning time of 40–60 min each. SPECT data is displayed in colour scale and CT data is in grey scale, respectively (high to low signals: top to bottom).

Tumour-bearing SCID/Beige mice were imaged by SPECT with computed tomography (CT) scanning to study the organ bio-distribution of radiolabelled L and t-L. The mice were inoculated with either the two melanoma or the two pancreatic human xenograft models on their back left flank (αvβ6 negative tumour: A375Ppuro or PANC-1) (right of the image) or right flank (αvβ6 positive tumour: A375Pβ6 or PANC0403) (left of the image). They were imaged immediately after injection, and 4 and 24 h post-injection by SPECT/CT. As shown in Fig. 4, no differences in the whole body bio-distribution for [^111^In]L and [^111^In]t-L were observed. At early time-points, good blood circulation was observed for both types of liposomes as indicated by signals seen in the heart and head of the mice. Over time, the liposomes accumulated in the liver and spleen. Slight accumulation of the liposomes in the tumours could also be seen at later time-points, but this could not be clearly observed in these images, possibly due to masking by prolonged blood circulation of the liposomes. L and t-L gave comparable signals in both the positive and negative tumours. Gamma counting was used to quantify the tumour uptake of L and t-L.

### 3.7 Comparable tumour distribution for both L and t-L was obtained in integrin negative and positive models

**Fig. 5:**
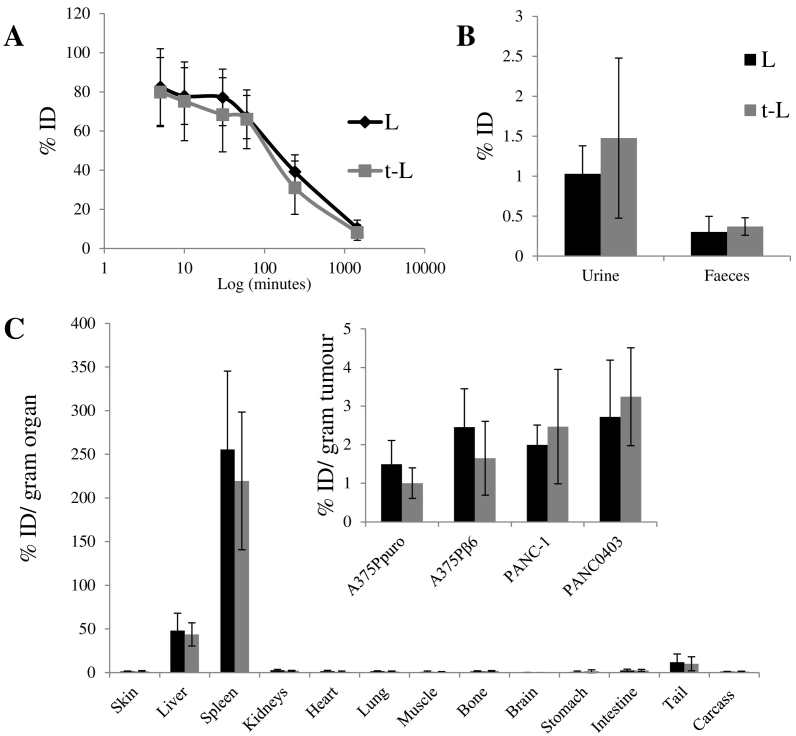
*In vivo* bio-distribution of radiolabelled L and t-L in tumour bearing SCID/Beige mice after single dose administration *via* tail vein injection. (A) Blood clearance profile of liposomes expressed as %ID. (B) Excretion profile of liposomes after 24 h expressed as %ID. (C) Results were expressed as percentage injected dose per gram of organ (%ID/g organ) at 24 h after injection of 2 μmol liposome/mouse. Organ biodistribution data were taken from mice bearing A375Ppuro and A375Pβ6 tumours. No significant difference between the bio-distribution of the untargeted and targeted liposomes was seen. Data was expressed as means ± SD (*n* = 3).

The organ bio-distribution and tumour uptake of [^111^In]L and [^111^In]t-L in tumour-bearing SCID/Beige mice, was then quantitatively determined by gamma counting. No significant differences were observed in the blood circulation profiles of [^111^In]L and [^111^In]t-L (Fig. 5A). At 1 h post administration, ~ 66%ID of both formulations remained in the blood, decreasing to 31–39%ID and ~ 8%ID after 4 and 24 h, respectively. L and t-L also had similar excretion profiles (Fig. 5B), with only small fraction of the liposomes excreted within 24 h in the urine (1.0–1.5%ID) and faeces (0.30–0.37%ID). In agreement with the SPECT imaging studies, liposomes accumulated mainly in the liver (L: 43 ± 3 %ID/g t-L: 29 ± 9 %ID/g) and the spleen (L: 255.5 ± 89.7 %ID/g t-L: 219.4 ± 78.8 %ID/g) (Figs. 5C and S8). No significant differences in the organ bio-distribution of [^111^In]L and [^111^In]t-L were observed. Additionally, no difference in uptake of t-L and L was observed in the αvβ6 positive tumours. A comparison of the uptake of both [^111^In]L and [^111^In]t-L among the different tumour models showed that when the tumours were normalised by weight, there was no significant difference in the uptake of the liposomes among different tumour models (1.7–3.2 %ID/g tumour). Once again no difference in bio-distribution was observed between [^111^In]L and [^111^In]t-L.

### 3.8 Both L-ALD and t-L-ALD sensitise tumours to γδ T cell therapy *in vivo*

**Fig. 6:**
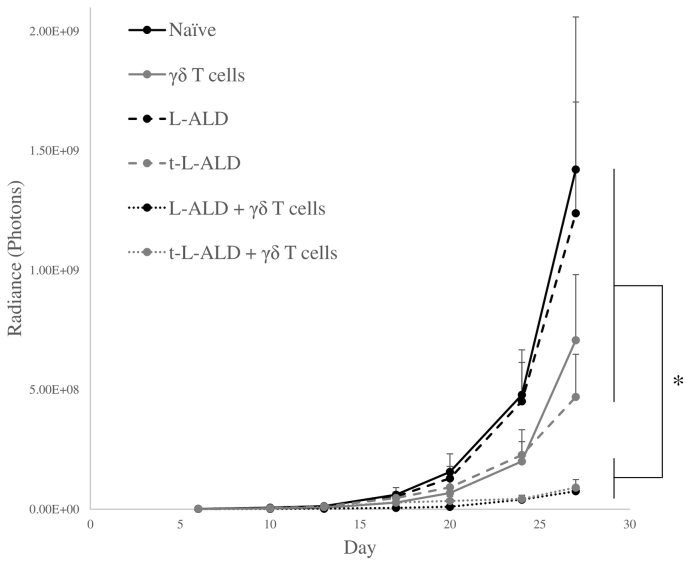
*In vivo* tumour therapy study. Experimental metastatic lung A375Pβ6 tumour bearing mice were treated intravenously on day 6 with L-ALD, t-L-ALD (0.5 μmol ALD/mouse), 1 × 10^7^ γδ T cells/mouse or were pre-treated with L-ALD or t-L-ALD 24 h prior to injection of γδ T cells. Three similar treatments were given intravenously at one week intervals, commencing on day 6 post-tumour inoculation. Tumour progression was monitored by bioluminescence imaging. A significant reduction in tumour growth was observed for the L-ALD/γδ and t-L-ALD/γδ combinatory immunotherapy groups compared to control mice or those treated with monotherapy of γδ T cells, L-ALD or t-L-ALD. t-L-ALD monotherapy resulted in an inhibition of tumour growth but this was not significant (*p* = 0.15). Data was expressed as mean ± SEM (*n* = 7). *p < 0.05, (ANOVA).

L-ALD and t-L-ALD were used as a monotherapy and in combination with *ex vivo*-expanded Vγ9Vδ2 T cells in an experimental metastatic lung model with the αvβ6 positive A375Pβ6 melanoma cell line in NOD-SCID gamma (NSG) mice. On day 6, all six groups had the same average tumour size (~ 1.3 × 10^6^ photons, as determined by bioluminescence imaging). L-ALD, t-L-ALD or γδ T cells as monotherapies did not result in a significant reduction in tumour growth. Monotherapy with t-L-ALD showed a trend towards greater effectiveness than L-ALD (4.70 × 10^8^ ± 1.78 × 10^8^ *vs.* 1.24 × 10^9^ ± 4.65 × 10^8^ photons, day 27, *p* = 0.15). However, this difference was not significant due to variability in tumour size. Mice pre-treated with L-ALD or t-L-ALD 24 h prior to injection of γδ T cells showed a significant reduction in tumour growth, with tumour sizes of 7.53 × 10^7^ ± 2.02 × 10^7^ and 9.06 × 10^7^ ± 3.33 × 10^7^ photons, respectively, compared to 1.42 × 10^9^ ± 6.38 × 10^8^ photons for naïve tumours on day 27 (Fig. 6). No improvement on the ability of t-L-ALD to sensitise the tumours to γδ T cells compared to L-ALD was observed. IFN-γ serum levels were measured on day 27. Mice pre-treated with L-ALD or t-L-ALD prior to γδ T cells had levels of 32.6 ± 19.3 and 12.3 ± 4.4 pg/ml (*p* < 0.05), respectively, compared to only 6.5 ± 0.9 pg/ml in γδ T cells-only treated group, mirroring the significant reduction in tumour growth observed in these groups.

### 3.9 Use of mouse sera in cell uptake studies reduces receptor-mediated cell uptake of t-L *in vitro*

**Fig. 7:**
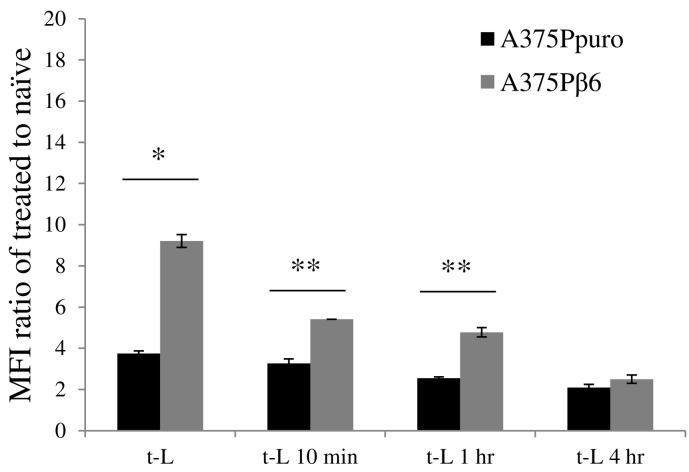
Effect of incubation of t-L with mouse serum on targeting efficacy. t-L were exposed to mouse serum for various lengths of times (10 min, 1 h, 4 h). The melanoma cell lines, A375Ppuro and A375Pβ6 were incubated with these liposomes for 1 h. Results are displayed as the MFI fold increase of naïve cells. Incubation of t-L with mouse serum leads to a reduction in receptor-mediated uptake cell uptake in the αvβ6 positive A375Pβ6 cell line. Cellular uptake was assessed by measuring the MFI using flow cytometry and FL-1 detector. Values are expressed as mean ± SD (*n* = 3). *p < 0.05, **p < 0.01, (Student's *t-*test A375Ppuro *vs.* A375Pβ6).

In order to explore one possible explanation for the lack of improvement in therapeutic efficacy of t-L-ALD compared to L-ALD when used in combination with γδ T cells in therapy studies, cell uptake studies were performed *in vitro*, whereby t-L were pre-incubated with mouse serum and excess serum and unbound proteins were removed by size exclusion chromatography, prior to their 1 h incubation with cell lines (Fig. 7). Interestingly, this study showed the reduction in receptor-mediated uptake was indeed dependent on the incubation times with mouse serum. This result suggests that mouse serum played a direct role in reducing active targeting efficiency *in vivo*. Unfortunately, whether this was a direct effect of the enzymes present in the serum on A20FMDV2 peptide's stability or a protein corona effect, could not be precisely established.

## 4 Discussion

In our study, αvβ6 positive cells were successfully targeted *in vitro*. Higher uptake of t-L *versus* L was observed in the A375Pβ6 and PANC0403 αvβ6 positive cell lines. This increased uptake of t-L led to an increased therapeutic efficacy of t-L-ALD at sensitising A375Pβ6 to γδ T cells when compared to L-ALD. The amount of receptor present on the surface of cells is a key consideration when evaluating a targeted drug delivery system. The expression of αvβ6 receptor on A375Pβ6 cells was confirmed *in vivo* in a flank mouse tumour model in a previous study carried out by co-authors of this paper. SPECT/CT imaging showed that the radiolabelled peptide A20FMDV2 had seven times higher retention in the αvβ6 positive A375Pβ6 solid tumours than the αvβ6 negative A375Ppuro tumour implanted in the same mouse (Saha et al., 2010). This demonstrates the expression of the αvβ6 receptor *in vivo*. In our study, it has been shown that the targeting efficiency of t-L was linked to αvβ6 integrin receptor expression on the cell surface. As expected, the targeting efficiency was directly proportional the level of receptor expression, when comparing the three αvβ6 positive cell lines with A375Pβ6 > PANC0403 > 4T1 in terms of both αvβ6 integrin receptor expression and targeting efficiency. This correlation has been previously reported by Elias et al. [33], with the density of the cell-surface HER2/neu receptor shown to be an important factor in the targeting capability of HER2-targeted nanoparticles.

While the peptide A20FMDV2 has previously been shown to selectively target the integrin receptor αvβ6 *in vivo* [12], [13], [34], it has not been conjugated to a nanoparticle before. In this work we provide evidence of successful conjugation of the peptide A20FMDV2 to the surface of liposomes for the first time, and have shown using *in vitro* peptide inhibition studies that the t-L are taken up by cells in a receptor-specific manner. Only one study by Grey et al. has specifically targeted the αvβ6 integrin receptor using a liposomal formulation [14]. In this study, liposomes were conjugated to the αvβ6-specific peptide, H2009.1. Interestingly, authors have shown that *in vitro* targeting using the αvβ6-positive cell line, H2009, could be achieved only when the peptide was incorporated in the tetrameric form and not the monomeric form. Our results have shown that *in vitro* targeting could be achieved despite using the monomeric form of the peptide. Future work investigating the use of multivalent forms of the peptide A20FMDV2 to further increase targeting efficiency is therefore worth investigating.

In our study, while active targeting could be achieved *in vitro* using t-L, no increase in %ID/g tumour, could be seen in αvβ6 positive tumours during *in vivo* bio-distribution analysis. There have been previous reports in the literature demonstrating that peptide-targeted liposomes showed no improvement in tumour accumulation compared to untargeted liposomes *in vivo* [35]. It was therefore not surprising to see comparable tumour uptake data *in vivo* for L and t-L in all tumours tested. However, we hypothesised that targeting L-ALD to the αvβ6 integrin receptor, known to be overexpressed on cancer cells and absent on healthy cells, will result in increased uptake of t-L-ALD due to receptor-mediated endocytosis *in vivo* and hence better therapeutic outcomes, when combined with γδ T cell-based immunotherapy. Kirpotin et al. demonstrated that despite no increase in tumour uptake of HER2 targeted liposomes in subcutaneous HER-2 positive breast cancer tumours *in vivo*, when excised tumours were studied using flow cytometry and microscopy, a 6-fold increase in cell internalisation of these targeted liposomes was observed [36]. Similarly, in another study, transferrin-targeted siRNA nanoparticles did not show an increase in tumour accumulation but resulted in a ~ 50% decrease in tumour growth compared to non-targeted nanoparticles due to increased cellular uptake [37]. Our therapy in experimental metastatic melanoma A375Pβ6-lung model results showed no added advantage using t-L-ALD over L-ALD when used in combination with γδ T cells, following our treatment protocols. There could be several explanations to the results obtained; the most obvious one is that L-ALD, in combination with immunotherapy, resulted in efficient tumour growth delay, decreasing the scope for any further improvements, using the targeted delivery approach to be detected. Using lower doses of L-ALD and t-L-ALD may reveal a difference in therapeutic efficacy of the two formulations. Additionally, when L-ALD and t-L-ALD were used as monotherapies, mice treated with t-L-ALD had an average tumour size that was 2.6 times lower than that of mice treated with L-ALD. We hypothesise that this may be as results of higher affinity of t-L-ALD than L-ALD to A375Pβ6 cells as demonstrated during *in vitro* studies. Another possibility could be that ALD experiences better endosomal escape from t-L-ALD than L-ALD, facilitated by the receptor-mediated uptake of the former. Unfortunately however, large variations in tumour size meant that this reduction in tumour size was not significant. Using higher doses of L-ALD and t-L-ALD as monotherapies or increasing the number of mice per treatment group may allow the difference in therapeutic efficacy to be observed.

The lack of correlation between the *in vitro* and *in vivo* targeting efficacy that we observed agrees with a previous study by Gray et al. [14]. As discussed above, liposomes targeted using the αvβ6-specific peptide H2009 in its tetrameric form demonstrated encouraging findings *in vitro*, with a 5–10 fold increase in uptake for targeted liposome. However, targeted liposomes did not lead to an improvement in targeting or efficacy *in vivo* using an αvβ6 positive H2009 subcutaneous tumour model [35]. Gray and his colleagues, concluded that it is the properties of the liposome (size and PEGylation) and not the targeting peptide that determines *in vivo* bio-distribution. Poor tumour penetration due to the dense extracellular matrix and high interstitial pressure in solid tumours may have prevented liposomes from exerting a targeting effect within the tumour itself [38], [39]. Likewise, a folate-targeted formulation of L-ALD demonstrated *in vitro* targeting but no therapeutic advantage was observed during *in vivo* studies with a folate receptor-α positive ovarian tumour model [24]. The presence of folate receptors on endogenous non-tumour cells leading to accelerated systemic clearance was hypothesised to be responsible for the lower therapeutic efficacy of the targeted formulation.

Recent studies have revealed that the formation of a protein corona on the surface of liposomes can lead to an inhibition of targeting efficacy, as the targeting-peptides can be hidden from their receptors [40], [41], [42], [43]. We have therefore conducted a study to see if incubation of t-L-ALD with mouse serum will compromise the targeting efficiency, in an attempt to correlate the *in vitro* targeting efficiency with *in vivo* active targeting in a living mouse. Our results indeed showed that incubation with mouse serum reduced the targeting efficiency of t-L, in a time-dependent manner. This suggests that the direct interaction of mouse serum and t-L adversely affects active targeting of t-L-ALD. Further studies to explore types of protein corona involved are warranted and as it has been reported that protein corona may differ based on what species the serum is from, extrapolation of data in mice to humans is not possible [40]. Another explanation of the mouse serum data could be the enzymatic degradation of A20FMDV2. A study performed by Saha et al. has shown that only 50% of the peptide A20FMDV2 [13] remained intact after 4 h incubation with mouse serum. Amino acid modifications may help improve resistance to enzymatic degradation [44], [45], [46]. To understand the exact effect of serum on active targeting in our study needs further investigation.

## 5 Conclusion

In this work, A20FMDV2 peptide was successfully conjugated to PEGylated liposomes and active targeting was confirmed *in vitro* in melanoma and pancreatic cells lines. *In vitro* targeting efficiency was directly linked to levels of αvβ6 receptor expressions and was blocked by addition of excess peptide, suggesting receptor-mediated endocytosis as a mechanism of internalisation. αvβ6-targeted liposomal alendronate led to significant improvement in sensitisation of αvβ6 positive cancer cell line to γδ T cells, and improved cell kill *in vitro*. Despite the slight promise in using t-L-ALD as a monotherapy, no added advantage was observed when combined with γδ T cells immunotherapy, in an experimental metastatic lung mice model. Future therapy studies with modified treatment protocols need to be performed to determine if a significant improvement in therapeutic efficacy using t-L-ALD can be achieved. *In vivo* peptide stability and the protein corona effect may be responsible for the decrease in *in vivo* targeting efficacy compared to the *in vitro* results obtained, which also needs to be investigated further.

## Appendix A Supplementary data

Supplementary material Image 1
