# Supplementary material for: Investigating in vitro and in vivo αvβ6 integrin receptor-targeting liposomal alendronate for combinatory γδ T cell immunotherapy
Source: J Control Release. 2017 Jun 28;256:141–52. doi: 10.1016/j.jconrel.2017.04.025 (PMC5488751; doi:10.1016/j.jconrel.2017.04.025)
Supplement: Supplementary file 1 — Supplementary material [file mmc1.docx]

**Investigating *in vitro* and *in vivo* αvβ6 integrin receptor-targeting liposomal alendronate for combinatory γδ T cell immunotherapy**

Naomi O. Hodgins, Wafa’ T Al-Jamal, Julie T-W. Wang, Rebecca Klippstein, Jane K. Sosabowski, John F. Marshall, John Maher and Khuloud T. Al-Jamal*

N.O. Hodgins, Dr. J. Wang, Dr. R. Klippstein, Dr. K.T. Al-Jamal

King’s College London

150 Stamford Street

Institute of Pharmaceutical Science

Franklin-Wilkins Building

150 Stamford Street

London SE1 9NH, UK

Dr. W.T. Al-Jamal

School of Pharmacy,

University of East Anglia, Norwich Research Park

Norwich NR4 7TJ, UK

Dr. Jane Sosabowski

Barts Cancer Institute

Queen Mary University of London

London EC1M 6BQ, UK

Prof. J.F. Marshall

Centre for Tumour Biology,

Barts Cancer Institute,

Queen Mary University of London,

London, EC1M 6BQ, UK

Dr. J. Maher

King’s College London

Division of Cancer Studies

Guy’s Hospital

London SE1 9RT

* Corresponding author

Prof. Khuloud T. Al-Jamal

Chair of Drug Delivery & Nanomedicine

Institute of Pharmaceutical Science, King’s College London, Franklin-Wilkins Building,

150 Stamford Street, London SE1 9NH, UK. Tel: +44(0)20-7848-4525

Email: khuloud.al-jamal@kcl.ac.uk

Keywords: integrin targeting, bisphosphonates, γδ T cells, liposomes, immunotherapy.

**SUPPLEMENTARY MATERIALS**

Human AB serum (male), ferric chloride hexahydrate, ammonium thiocyanate (S1120), copper (II) sulphate pentahydrate were purchased from Sigma (UK). Ficoll-Paque Plus was purchased from GE Healthcare (UK). Citrate-Dextrose Solution was purchased from SLS (UK). Human AB serum (male) was obtained from Sigma (UK). IL-2 (100U/ml) (Proleukin) was obtained from Prometheus (USA). Zoledronic acid was obtained from Novartis (Switzerland). TCR Pan gamma/delta-FITC and IgG FITC Isotype control was purchased from Beckman Coulter (UK). All reagents were used without further purification.

**SUPPLEMENTARY METHODS**

**Physicochemical characterisation of liposomes**

The hydrodynamic diameter, polydispersity index and Zeta potential of the liposomes were measured using the NanoZS (Malvern Instrument, UK). Hydrodynamic size, polydispersity index and Zeta potential were measured in disposable square polystyrene cuvettes and disposable capillary cells, respectively (Malvern Instrument, UK). The sample (20 µl) was diluted to 1.5 ml with 10 mM Sodium Chloride. The measurements were carried out at room temperature. Three measurements were performed and the mean and standard deviation were calculated for each sample.

**Lipid recovery quantification**

Stewart’s assay was used to determine the lipid concentrations before and after purification with size exclusion chromatography [[1](#_ENREF_1)]. Stewart’s reagent (0.1 M ammonium ferrothiocyanate solution) was prepared using 5.46 g ferric chloride hexahydrate and 6.08 g of ammonium thiocyanate and made up to 200 ml with deionised water. The solution to be measured was prepared by mixing 50 μl of the unknown sample (or the standard), 2 ml of chloroform and 2 ml of Stewart’s Reagent. The mixture was centrifuged at 1000 g in a bench centrifuge (Centrifuge 5810 R, Eppendorf) for 10 min and the organic layer was removed and analysed by UV spectrometer (Lambda 35, Perkin Elmer). The absorbance at 485 nm was used to determine the lipid concentration. Calibration curves were prepared in the same way using known amounts of lipid as standards. The lipid recovery was determined by comparing the lipid concentration before and after purification.

**Quantification of ALD**

ALD concentrations, for determination of % EE, were determined with a copper sulphate-based UV detection method [[2](#_ENREF_2)]. A calibration curve containing 5 mM empty liposomes and between 0.1-1 mM of free ALD, referred to ‘ALD spiked liposomes’ samples, was prepared. L-ALD samples to be quantified (or standards) were both processed using the Folch method prior to quantification. This method disrupts the liposomes; allowing the encapsulated ALD to be released into the aqueous phase and separating any hydrophobic components (cholesterol, lipids etc.) from the hydrophilic drug. In brief, chloroform and methanol were added to a sample of liposomes at 8:4:3 (chloroform: methanol: liposomes) volume ratio. The sample was then vortexed (Vortex genie 2, Scientific Industries Inc, USA) and centrifuged at 10000 rpm for 10 minutes (Centrifuge 5810 R, Eppendorf). Two layers were formed after centrifugation and the upper aqueous layer containing the ALD was removed and quantified. CuSO_4_ reagent was prepared by dissolving 10 mM CuSO_4_ in deionised water. A 0.5 ml sample of the upper aqueous phase was to 0.5 ml of 10 mM CuSO_4_ reagent. After 10 minutes, the UV absorbance at 240 nm was measured with a UV/Vis Spectrophotometer (Perkin Elmer, Model: Lambda 35), using HBS as the reagent blank. Concentrations were calculated from the equation obtained from the ‘ALD spiked liposomes’ calibration curve. Encapsulation Efficiency (EE %) was calculated by comparing the ZOL and ALD concentration of liposomes to the concentration before purification. The quantity of N-BP in each liposome sample was measured three times and expressed as mean ± standard.

**Isolation and expansion of Vγ9Vδ2 T cells**

Blood samples of 20-30 ml were obtained from healthy volunteers. Ethical approval, “Use of Donor Blood Samples for Pre-Clinical Development of Active and Passive Immunotherapy for Cancer” (Ref.09/H0804/92) was obtained. The blood sample was added to 5 ml of Citrate-Dextrose Solution to prevent clotting. The sample was then layered on top of 15 ml of Ficoll-Paque Plus and centrifuged at 1150 g for 25 min, with no acceleration or breaks, using a bench centrifuge (Centrifuge 5810 R, Eppendorf). The layer of cells between the Ficoll-Paque Plus and the plasma was then removed and the resulting PBMCs were washed twice with PBS and were then suspended in RPMI 1640 (containing 10% human AB serum, 1% Glutamax and 1% antibiotic-antimycotic solution) at a concentration of 3x10^6^ cells/ml. In order to expand the γδ T cells, the PBMCs were activated with 1 µg/ml ZOL and 100 U/ml IL-2. Additional medium and 100 U/ml IL-2 were added every 2-3 days for 15 days.

**Flow cytometry analysis of γδ T cells**

On Day 1 and Day 15, 200 µl samples of the cell suspension were taken and 5 µl of either T Cell Receptor (TCR) Pan γ/δ-FITC antibody or IgG1 FITC Isotype control antibody was added. The cells were incubated with the antibodies for 20 min at 4°C before 1 ml PBS was added. Cells were centrifuged at 1000 rpm for 5 min in a bench centrifuge (Centrifuge 5810 R, Eppendorf). The supernatant was discarded and the cell pellet was re-suspended in 500 µL of PBS. All flow cytometric data were acquired using a Beckman Coulter Cytometer FC 500 MPL and were analysed using CXP Analysis software (Beckmann Coulter). The lymphocyte cell population was gated and the number of cells in this gate that express the γδ TCR were calculated as a percentage of the total lymphocytes.

**REFERENCES**

[1] R. New, Liposomes: A Practical approach, (1990).

[2] M. Koba, K. Koba, L. Przyborowski, Application of UV-derivative spectrophotometry for determination of some bisphosphonates drugs in pharmaceutical formulations, Acta poloniae pharmaceutica, 65 (2008) 289-294.

**SUPPLEMENTARY FIGURES**

**Figure S1: Separation of liposomes from peptide by size exclusion chromatography. (A)** Calibration curve obtained for the peptide A20FMDV2 using the LavaPep Peptide Quantification Kit. **(B)** A Sepharose 2B column was used to separate the unreacted peptide from the liposomes. A physical mixture of untargeted liposomes and free peptide was initially loaded onto the column. Each fraction eluted from the column was tested for lipid and peptide content by Stewert’s assay and a peptide quantification kit (LavaPep), respectively. Distinct elution profiles of the two components were obtained.

**Table S1: Physicochemical characteristics of liposomes formulated in this study.**

| **DSPE-PEG2000- Maleimide (%mol)** | **Initial peptide loading**  **(µg/µmol lipid)** | **Size (nm) ^[a], [c]^** | **PDI^[a], [c]^** | **Zeta Potential (mV) ^[b], [c]^** |
| --- | --- | --- | --- | --- |
| **0.5%** | 0 | 159.9 ± 2.433 | 0.054 ± 0.008 | -11.7 ± 0.862 |
|  | 25 | 157.6 ± 3.156 | 0.061 ± 0.033 | -10.9 ± 0.173 |
| **2%** | 0 | 152.2 ± 1.572 | 0.046 ± 0.011 | -9.28 ± 0.286 |
|  | 25 | 150.2 ± 2.616 | 0.056 ± 0.011 | -11.1 ± 0.794 |

[a] Hydrodynamic diameter measured by dynamic light scattering.

[b] Analysed by electrophoretic light scattering using 10mM NaCl.

[c] Data are represented as mean ± SD (n=3).

**Table S2: Optimisation of peptide loading on liposomes.**

| **DSPE-PEG2000- Maleimide (%mol)** | **Initial peptide loading**  **(µg/µmol lipid)** | **Final peptide loading**  **(µg/µmol lipid) [a] , [c]** | **nmol peptide/µmol lipid [a] , [c]** | **% Peptide conjugated [b], [c]** |
| --- | --- | --- | --- | --- |
| 0.5 % | 50 | 12.0 ± 2.0 | 4.7 ± 0.8 | 11.7 ± 1.95 |
| 2 % | 50 | 51.6 ± 10.2 | 20.2 ± 4 | 50.31 ± 9.9 |

[a] Determined by LavaPep Peptide Quantification Kit.

[b] Calculated as a percentage of initial peptide added.

[c] Data are represented as mean ± SD (n-3).

**Figure S2: Effect of %mol DSPE-PEG 2000 maleimide on the cellular uptake of liposomes*.*** The melanoma cell lines, A375Ppuro (αvβ6 -ve) and A375Pβ6 (αvβ6 +ve), were incubated with L or t-L containing 1 mol% DSPE-PEG_2000_ maleimide for either 1 or 4 h. Cellular uptake was assessed by measuring the MFI using flow cytometry and FL-1 detector. Results are displayed as the MFI ratio of t-L compared to L. Liposomes containing 1% DSPE-PEG_2000_ maleimide showed a significant targeting effect at 1 and 4 h. Values are expressed as mean ± SD (n= 4). **P* < 0.05, ***P <* 0.01, ****P* < 0.001, (Student’s *t* test A375Ppuro vs. A375Pβ6).

**Figure S3: Effect of %mol DSPE-PEG 2000 maleimide on the cellular uptake of liposomes*.*** The melanoma cell lines, A375Ppuro (αvβ6 -ve) and A375Pβ6 (αvβ6 +ve), were incubated with L or t-L containing 0.5 or 2 % mol DSPE-PEG2000 maleimide for either 1 or 4 h. Cellular uptake was assessed by measuring the MFI using flow cytometry and FL-1 detector. Results are displayed as the MFI ratio of t-L compared to L. t-L showed significant targeting ability at 1 h, but at 4 h this targeting effect was no longer observed. Values are expressed as mean ± SD (n= 4). **P* < 0.05, ***P <* 0.01, ****P* < 0.001, (Student’s *t* test A375Ppuro vs. A375Pβ6).

**Figure S4: Effect of serum on the cellular uptake of liposomes*.* (A)** A375Ppuro (αvβ6 -ve) and A375Pβ6 (αvβ6 +ve)cells were incubated with L or t-L in the presence or absence of serum for either 1 or 4 h. Cellular uptake was assessed by measuring the MFI using flow cytometry and FL-1 detector. Results are displayed as the MFI fold increase of the naïve. When no serum was present, much higher uptake of both L and t-L occurred **(B)** A375Ppuro and A375Pβ6 cells were incubated with L or t-L in the absence of serum for either 1 or 4 h. Results are displayed as the MFI ratio of t-L compared to L. The targeting effect of t-L compared to L was no longer seen. Values are expressed as mean ± SD (n= 4).

**Figure S5: Cellular uptake of liposomes in αvβ6 negative cancer cell lines *in vitro.*** A375Ppuro and PANC-1 cells were incubated with L or t-L labelled with 1mol% CF-DOPE for either 1 or 4 h. Cells were pre-treated on ice with either normal media or media containing 50 μg/ml of the free A20FMDV2 peptide for 10 min prior to addition of the liposomes. **(A)** Cellular uptake was assessed by measuring the mean fluorescence intensity (MFI) using flow cytometry and FL-1 detector. Sample graphs shown of incubation of L and t-L with A375Ppuro cells. **(B)** Unexpectedly, at some concentrations, incubation with free A20FMDV2 did reduce uptake of t-L. This reduction was smaller than that seen in αvβ6 positive cancer cell lines. **(C)** In PANC-1 cells, uptake of t-L is unaffected by the presence of free A20FMDV2. **P* < 0.05, ***P <* 0.01, ****P* < 0.001 (Student’s *t* test +A20FMDV2 vs. –A20FMDV2)​

**Figure S6: The ability of L-ALD and t-L-ALD to activate γδ T cells. (A)** Cells were treated with ALD, L-ALD or t-L-ALD for 24 hours at 30 or 60 µM for 24 h. The treatments were then removed and replaced with 2 x 10^5^ γδ T cells for a further 24 h before a MTT assay was performed. t-L-ALD did not increased the sensitivity of A375Ppuro cells to γδ T cells. ALD, L-ALD or t-L-ALD did not cause cytotoxicity alone. The liposomal formulation was not as effective as the free drug. **(B)** IFN**-**γ ELISA was performed on supernatant removed prior to the MTT assay. Data was expressed as means ± SD (n=5)​(Student’s *t* test L-ALD vs. t-L-ALD).

**Figure S7: Radiolabelling efficiency of liposomes with ^111^In and radiolabelling stability in serum. (A)** TLC of the radiolabelled liposomes immediately after radiolabelling. (**B)** TLC of purified radiolabelled liposomes after incubation with either FBS or PBS for 24 h at 37°C. Free **^1^**^11^In was removed by passing the liposomes through a PD10 desalting column. L exhibited better labelling and stability than t-L.

**Figure S8: *In vivo* Biodistribution of radiolabelled L and t-L in tumour bearing mice after single dose administration *via* tail vein injection.** Results were expressed as percentage injected dose per organ (%ID/organ) at 24 h after injection of 2 μmol liposome/mouse in tumour bearing SCID/Beige mice. Data was expressed as means ± SD (*n*=3). **P* < 0.05, ***P <* 0.01, ****P* < 0.001 (Student’s t test L vs. t-L, statistical significance among tumour models determined by ANOVA).
